# Quantifying temporal change in plant population attributes: insights from a resurrection approach

**DOI:** 10.1093/aobpla/ply063

**Published:** 2018-10-10

**Authors:** Rocío Gómez, Belén Méndez-Vigo, Arnald Marcer, Carlos Alonso-Blanco, F Xavier Picó

**Affiliations:** 1 Departamento de Ecología Integrativa, Estación Biológica de Doñana (EBD), Consejo Superior de Investigaciones Cientíﬁcas (CSIC), Sevilla, Spain; 2 Departamento de Genética Molecular de Plantas, Centro Nacional de Biotecnología (CNB), Consejo Superior de Investigaciones Cientíﬁcas (CSIC), Madrid, Spain; 3 CREAF, Cerdanyola del Vallès, Spain; 4 Universitat Autònoma de Barcelona, Cerdanyola del Vallès, Spain

**Keywords:** *Arabidopsis thaliana*, broad sense heritability, field experiments, flowering time, microsatellite genotyping, precipitation, resurrection approach, warming

## Abstract

Rapid evolution in annual plants can be quantified by comparing phenotypic and genetic changes between past and contemporary individuals from the same populations over several generations. Such knowledge will help understand the response of plants to rapid environmental shifts, such as the ones imposed by global climate change. To that end, we undertook a resurrection approach in Spanish populations of the annual plant *Arabidopsis thaliana* that were sampled twice over a decade. Annual weather records were compared to their historical records to extract patterns of climatic shifts over time. We evaluated the differences between samplings in flowering time, a key life-history trait with adaptive significance, with a field experiment. We also estimated genetic diversity and differentiation based on neutral nuclear markers and nucleotide diversity in candidate flowering time (*FRI* and *FLC*) and seed dormancy (*DOG1*) genes. The role of genetic drift was estimated by computing effective population sizes with the temporal method. Overall, two climatic scenarios were detected: intense warming with increased precipitation and moderate warming with decreased precipitation. The average flowering time varied little between samplings. Instead, within-population variation in flowering time exhibited a decreasing trend over time. Substantial temporal changes in genetic diversity and differentiation were observed with both nuclear microsatellites and candidate genes in all populations, which were interpreted as the result of natural demographic fluctuations. We conclude that drought stress caused by moderate warming with decreased precipitation may have the potential to reduce within-population variation in key life-cycle traits, perhaps as a result of stabilizing selection on them, and to constrain the genetic differentiation over time. Besides, the demographic behaviour of populations probably accounts for the substantial temporal patterns of genetic variation, while keeping rather constant those of phenotypic variation.

## Teaser

Plant populations vary over time as a result of the effects of environmental variation on life-cycle traits and genetic diversity. It is important to quantify how much populations actually change over time to better understand the response of plants to rapid environmental shifts. Our resurrection approach indicated that populations of the annual plant *Arabidopsis thaliana* exhibited substantial genetic changes over just a decade. Interestingly, such changes seemed to be mediated by the combination of the extent of warming and precipitation. Nevertheless, populations remained viable over time suggesting that plants may possess the means to cope with global climate change.

## Introduction

All plant populations exhibit to some extent spatio-temporal phenotypic and genetic changes as a result of environmental variation. The quantification of the pace and intensity of such phenotypic and genetic changes, as well as their impact on fitness and population persistence, is a particularly pressing issue to understand the evolutionary potential of plants in rapidly changing environments, such as the ones currently posed by global climate change (Shaw and Etterson 2012; Kopp and Matuszewski 2014). The dominant view of modern population genetics is that adaptation to changing environments is chiefly driven by the random genetic drift and selection on consistently beneficial or deleterious mutations (Messer *et al.* 2016). This paradigm implies a slow evolutionary process because common mutations are expected to have only small effects on fitness (Messer *et al.* 2016). However, important phenotypic changes, interpreted as events of rapid or contemporary evolution, have been observed in plant populations over relatively short periods of time for different plant traits, such as flowering time (Franks *et al.* 2007) and self-fertilization rates (Bodbyl Roels and Kelly 2011), as well as processes, such as the invasiveness of exotic species in recipient communities (Maron *et al.* 2004) or the dynamics of plant–pathogen interactions (Gilbert and Parker 2010). Overall, it is increasingly accepted that the standard genetic model might be insufficient to describe the rapid phenotypic adaptive changes observed in natural populations (Messer *et al.* 2016). Further work is clearly needed to quantify the timescales of genetic and phenotypic variation in plant populations and to infer their ecological and evolutionary consequences in a context of rapid environmental changes.

The resurrection approach represents a powerful, albeit time-consuming, means to quantify the extent of phenotypic and genetic variation in plant populations over time. In particular, the resurrection approach compares past and contemporary performance under common conditions of individuals, i.e. the ancestors and their descendants, whose seeds were collected from the same populations at different points in time encompassing several generations (Bennington *et al.* 1991; McGraw *et al.* 1991; Davis *et al.* 2005; Franks *et al*. 2007, 2008, 2018; Anderson *et al.* 2012; Franks and Hoffmann 2012; Nevo *et al.* 2012; Fukano *et al.* 2013; Gomulkiewicz and Shaw 2013; Sultan *et al.* 2013; Bustos-Segura *et al.* 2014; Van Dijk and Hautekèete 2014; Thomann *et al.* 2015; Welt *et al.* 2015; Etterson *et al.* 2016; Horgan-Kobelski *et al.* 2016; Kuester *et al.* 2016; O’Hara *et al.* 2016; Frachon *et al.* 2017). An exceptional value of the resurrection approach is that phenotypic differences in fitness-related traits between ancestors and descendants may be attributed to rapid evolution, i.e. fast genetically based evolutionary shifts imposed by environmental changes (Franks *et al.* 2007), which has been detected in annual plants over a few generations (Maron *et al.* 2004; Franks *et al.* 2007; Rhoné *et al.* 2010; Franks and Hoffmann 2012; Sultan *et al.* 2013; Etterson *et al.* 2016; Frachon *et al.* 2017). In order to enhance its impact and value, resurrection studies should address phenotypic variation in fitness-related traits along with neutral and functional genetic variation across generations. Whilst neutral genetic variation can provide hints on the demographic behaviour that populations experienced over time, functional genetic variation can provide insight into the genetic basis of variation in phenotypic traits.

Beyond the interest of the resurrection approach for evolutionary biology, resurrection experiments may also become extremely valuable in quantifying the responses of plant populations to climate change (Anderson *et al.* 2012; Franks *et al.* 2014; Etterson *et al.* 2016). Many climate change studies examine models predicting distribution range changes for a wide array of plants in climate change scenarios (Pearson and Dawson 2003; Beaumont *et al.* 2008; Bellard *et al.* 2012; Parmesan and Hanley 2015). Besides, there exists a large body of literature reporting generalized advances in plant phenology with a warming climate using existing long-term observational data sets (Peñuelas and Filella 2001; Menzel *et al.* 2006; Parmesan 2007; Parmesan and Hanley 2015), as well as estimating the effects of experimental warming on plant performance for various species in different biomes (Lloret *et al.* 2004; Walker *et al.* 2006; Wolkovich *et al.* 2012; Peñuelas *et al.* 2013). However, one important piece is still missing to fully comprehend the effects of climate change on plant populations and communities: the microevolutionary consequences of climate change, i.e. the genetic changes driven by selection imposed by environmental changes (Holt 1990; Karell *et al.* 2011). In the long term, the resurrection approach can supply this missing piece of knowledge by quantifying phenotypic and genetic variation across generations. Furthermore, if phenotypic variation in fitness-related traits between ancestors and descendants can be associated to variation in putative agents of natural selection that occurred between the points in time when seeds were collected, we will have the means to parameterize process-based models with functions affecting fitness under short- and mid-term climate change scenarios.

The main goal of this study was to quantify the extent of phenotypic and genetic temporal change in natural populations of the annual plant *Arabidopsis thaliana* by means of a resurrection approach. To this end, we used seeds from four well-known Spanish *A. thaliana* populations sampled in 2003–04 (Picó *et al.* 2008) and resampled in 2012–13 specifically for this study. Overall, these four populations are a good illustration of the Mediterranean therophyte community with a great diversity of annual plants including *A. thaliana*. We carried out a resurrection approach on *A. thaliana* through three specific objectives. First, we conducted a series of field experiments to estimate temporal variation in flowering time, a key developmental trait with adaptive significance affecting fitness in *A. thaliana* (Weinig *et al.* 2002; Korves *et al.* 2007; Ågren and Schemske 2012; Manzano-Piedras *et al.* 2014; Exposito-Alonso *et al.* 2018*a*). Besides, experiments were also performed to detect hidden genetic variation in flowering time in study populations over time. Second, we analysed the amount and spatio-temporal distribution of genetic variation by genotyping populations from the two samplings with neutral microsatellite loci, which provided hints on the demographic behaviour of *A. thaliana* populations over the study period (e.g. effective population size). Third, we quantified the spatio-temporal patterns of nucleotide diversity in well-known genes affecting life-history traits in populations from the two samplings. In particular, we sequenced *FRI* and *FLC*, two genes involved in the vernalization pathway for flowering (Koornneef *et al.* 1998; He *et al.* 2003; Kim *et al.* 2009; Kim and Sung 2014; Méndez-Vigo *et al.* 2016), and *DOG1*, a seed specific gene affecting seed dormancy (Alonso-Blanco *et al.* 2003; Bentsink *et al.* 2006, 2010; Kronholm *et al.* 2012; Chiang *et al.* 2013; Vidigal *et al.* 2016). Importantly, we overcame one of the main caveats of the resurrection approach, i.e. aging effects on stored seed collections, by simultaneously bulking up seeds from each population and sampling to remove aging and environmental maternal effects on stored old and field-collected contemporary seeds, respectively, prior to experiments. We discuss the results in the context of the added value and implications of the resurrection approach for evolutionary studies in plants in a rapidly changing world.

## Methods

### Source populations

**Figure 1.**
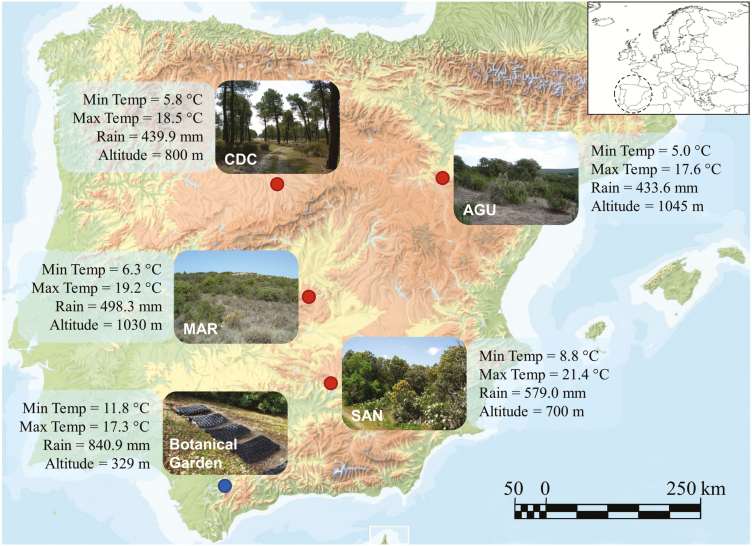
Map of Spain with the four *Arabidopsis thaliana* populations of study (red dots). A photograph of each population along with environmental characteristics of populations, such as average annual minimum temperature, average annual maximum temperature, total annual precipitation and altitude, are given. The location of the El Castillejo Botanical Garden where field experiments were conducted is also indicated (blue dot) along with a photograph of experimental blocks and the environmental characteristics of the experimental facility.

A total of four Spanish *A. thaliana* populations were selected for this study (Fig. 1): Aguarón (AGU; 41.32°N, 1.34°W, 1045 m a.s.l., Zaragoza province), Ciruelos de Coca (CDC; 41.21°N, 4.55°W, 800 m a.s.l., Segovia province), Marjaliza (MAR; 39.58°N, 3.93°W, 1030 m a.s.l., Toledo province) and Santa Elena (SAN; 38.33°N, 3.51°W, 700 m a.s.l., Jaén province). In these locations, *A. thaliana* occurs in typically Mediterranean environments (Fig. 1), such as forests and scrublands dominated by evergreen *Quercus* species (AGU, MAR and SAN) and sandy open sites and edges of *Pinus pinaster* forests (CDC). In all populations, *A. thaliana* occurred in patches of different size and density in the two samplings. Populations were also rather similar in terms of general weather conditions (Fig. 1), based on data available from different digital geographical databases used previously to characterize the Iberian collection of *A. thaliana* populations (Marcer *et al.* 2016). The four populations, on average separated by 267 km (range = 143.6–377.0 km), are genetically differentiated from each other (Picó *et al.* 2008; Méndez-Vigo *et al.* 2013).

All populations were sampled twice: AGU in 2004 and 2012 (spanning 8 years), CDC in 2003 and 2012 (9 years), MAR in 2003 and 2012 (9 years) and SAN in 2003 and 2013 (10 years). In the first and second samplings, we collected seeds from 20–32 and 60–70 individuals per population, respectively. In MAR, the individuals sampled in the second sampling were collected in 2012 (*N* = 29) and in 2013 (*N* = 41), but for the sake of simplicity we did not make any distinction between them. Important for this study, the two samplings were carried out around a GPS location recorded in the first sampling characterizing a representative abundant patch within each population. To the extent possible, we took the precaution to sample *A. thaliana* individuals separated from each other by at least 1 m within the same area around the GPS location (~50 × 50 m^2^) in the two samplings.

Field-collected seeds from the first sampling were multiplied by the single seed descent method in a glasshouse from the Centro Nacional de Biotecnología (CNB-CSIC) of Madrid a few months after collection. Multiplied seeds were stored in dry conditions in cellophane bags at room temperature in darkness, storing conditions that can preserve *A. thaliana* seeds for years. Collected seeds from the first sampling and field-collected seeds from the second sampling were multiplied again in the same manner in a single experiment in 2014 to obtain the final collection of seeds from individuals of the first (2003–04) and second (2012–13) sampling from each population.

### Environmental data

For each *A. thaliana* population, weather records were obtained from the Agencia Estatal de Meteorología of Spain (AEMET), including daily records of minimum and maximum temperatures and precipitation from the nearest automatic meteorological stations. We used data from 1–3 meteorological stations per population depending on their geographical distance to populations, as well as on the degree of data completeness for the study period. On average, meteorological stations were located at 28.9 km (range = 2.4–48.8 km) far from *A. thaliana* populations. When more than one meteorological station was used per population, i.e. MAR and SAN, we computed daily mean values across meteorological stations for minimum temperature, maximum temperature and precipitation. For each population, we also obtained historical climate data on average annual minimum temperature, average annual maximum temperature and total annual precipitation. Historical data were obtained from the Digital Climatic Atlas from the Iberian Peninsula, based on a spatial interpolation using records from a total of 2285 meteorological stations across Portugal and Spain during the period 1951–99.

Aerial orthophotographs were retrieved from different public administrations in Spain (i.e. Plan Nacional de Ortofotografía Aérea, Sistema de Información Territorial de Aragón, Portal de Mapas de la Junta de Comunidades de Castilla-La Mancha and Infraestructura de Datos Espaciales de Andalucía) and used to detect major landscape changes in *A. thaliana* populations over the study period **[see****Supporting Information—Fig. S1****]**. AGU, MAR and SAN did not show any significant major natural disturbance, e.g. fires or landslides, between samplings. In contrast, CDC exhibited some partial changes in tree density **[see****Supporting Information—Fig. S1****]**. Unlike the other populations, CDC occupies sandy open sites and edges of *P. pinaster* forests, which have been exploited for centuries for resin extraction. Nonetheless, *A. thaliana* distribution seemed not to be affected by this partial modification, probably because *A. thaliana* only occurred along *P. pinaster* forest edges. However, we ignore whether other biotic factors, e.g. herbivores and pathogens, have affected these populations over the time period of study. We can at least ensure that we did not detect any specific damage during the two samplings or in visits conducted between samplings to some of the populations, such as CDC and MAR (C. Alonso-Blanco and F. X. Picó, pers. obs.).

### Field experiments

We conducted a total of three field experiments using 15 haphazardly chosen individuals per population (CDC, MAR and SAN) and sampling (3 populations × 15 individuals × 2 samplings = 90 individuals). Due to technical problems, individuals from AGU from the 2012 sampling could not be multiplied and this population was excluded from the experiments. In addition, CDC and MAR from the first sampling were eventually represented by 14 individuals. Experiments were conducted at El Castillejo Botanical Garden of Sierra de Grazalema Natural Park in south-west Spain (Fig. 1) following the same protocols used in previous successful experiments with Iberian *A. thaliana* accessions at the same facility (Méndez-Vigo *et al.* 2013; Manzano-Piedras *et al.* 2014; Exposito-Alonso *et al.* 2018*a*). In short, all individuals were replicated six times in all experiments (3 experiments × 88 individuals × 6 replicates = 1584 experimental units). Each replicate contained 60 filled seeds (1584 experimental units × 60 seeds = 95040 seeds) that were sown in square plastic pots (12 × 12 × 12 cm^3^) filled with standard soil mixture (Abonos Naturales Cejudo Baena S.L., Utrera, Spain). Previously, batches of 60 seeds were prepared 1 month before each sowing, and stored in 1.5-mL plastic tubes at room temperature in darkness until the sowing day.

Experiments were designed to quantify flowering time in field conditions of *A. thaliana* individuals in three different scenarios determined by sowing dates. Hence, all individuals from each population and sampling were allowed to germinate in mid autumn (sowing date: 7 October 2014), late autumn (sowing date: 5 December 2014) and mid winter (sowing date: 27 January 2015). Such sowing schedule reproduced quite well the germination behaviour observed in natural populations from different Iberian environments (Montesinos *et al.* 2009; Picó 2012). Our goal was to force plants from the two samplings to complete the life cycle in progressively shorter periods of time as a means to express hidden genetic variation in flowering time (see Le Rouzic and Carlborg 2008). Although late spring germination is also observed in natural populations from Mediterranean environments, sowing in spring reduces the vegetative phase in a way that *A. thaliana* cannot complete the life cycle at El Castillejo Botanical Garden (F. X. Picó, unpubl. data). Flowering time in experimental field conditions was estimated as the number of days between 15 days after sowing, when most seeds had already germinated in all experiments, and flowering date. Flowering date was given at the pot level when the majority of plants in the pot, which were sisters and showed homogeneous flowering behaviour, had the first flower open. In these experiments, we only focused on flowering time because this trait showed the largest quantitative genetic differentiation among populations when compared with other life-cycle traits, e.g. recruitment and fecundity (Méndez-Vigo *et al.* 2013). Besides, flowering time estimated for *A. thaliana* in field conditions strongly correlates with fitness, estimated as the product between survivorship and fecundity (Exposito-Alonso *et al.* 2018*a*).

### Statistical analyses

For each experiment, general linear models were used to test the effect of sampling (fixed factor: 2003–04 and 2012–13), population (random factor: CDC, MAR and SAN) and individuals nested within population (random factor: 14–15 individuals per population) on flowering time. We inspected the distribution of the residuals to check that the assumptions of the analyses were met. For each population, sampling and experiment, we estimated the broad sense heritability of flowering time as *h*^2^ = *V*_G_/(*V*_G_ + *V*_E_), where *V*_G_ is the estimated among-individual variance component and *V*_E_ is the residual variance (Le Corre 2005). The 95 % confidence intervals (CIs) for *h*^2^ values were computed with the (co)variances method using restricted maximum likelihood (REML) variance components (Lynch and Walsh 1998). Analyses were performed with SPSS v.23 statistical software (IBM, Chicago, IL, USA).

### Genetic analyses

A total of 226 *A. thaliana* individuals were collected in the second sampling (2012–13). They were genotyped for 12 nuclear microsatellites (see DNA extraction and marker genotyping protocols in Picó *et al.* 2008; Méndez-Vigo *et al.* 2011). Individuals eventually used from the second sampling were distributed as follows: 53 from AGU, 58 from CDC, 70 from MAR and 45 from SAN. Microsatellite data from the second sampling and microsatellite data obtained from the first one (2003–04; Picó *et al.* 2008) were joined together. Microsatellite data obtained from the first sampling included a total of 103 individuals: 20, 32, 30 and 21 individuals from AGU, CDC, MAR and SAN, respectively. Hence, the overall microsatellite data set of our resurrection approach totalled 329 individuals. Ten individuals from the second sampling were genotyped twice for all microsatellites. This was used to estimate an average genotyping error rate of 0.021 per locus, similar in magnitude to the error obtained from the existing data set from the first sampling, i.e. 0.047 per locus (Picó *et al.* 2008). In both cases, microsatellite genotyping errors were mostly due to allele dropout at heterozygous loci. Electropherograms obtained for the microsatellite genotyping from the second sampling were visually inspected and manually scored using GeneMapper v.4.1 software (Applied Biosystems, Foster City, CA, USA).

The number of individuals genotyped per population in the second sampling more than doubled those from the first one. In order to avoid sample size effects and subsequent bias in genetic parameters, we created 100 random subsamples of 20 individuals each, which was the minimum sample size used in this study, per population and sampling. Genetic diversity was estimated with GenAlEx v.6.5 (Peakall and Smouse 2012), including observed heterozygosity (*H*_O_) and mean gene diversity (*H*_S_). We also computed outcrossing rates (*O*_R_) as (1 − *F*_IS_)/(1 + *F*_IS_), where *F*_IS_ is the inbreeding coefficient. For each population, genetic differentiation was also estimated by partitioning the genetic variance among samplings, among individuals within samplings and within individuals, calculating *F*-statistics via the analysis of molecular variance (AMOVA) with GenAlEx. Significance of *F*-statistics was estimated by performing 1000 permutations. Final values for genetic diversity and genetic differentiation parameters were obtained by calculating mean values across the 100 random subsamples of equal size. Finally, we computed the number and frequency of non-redundant multilocus genotypes (*N*_G_) per population and sampling, which provided a direct measure of unique combinations of alleles per population and sampling. We used *N*_G_ to perform principal coordinate analysis (PCoA) with GenAlEx for visualizing the relationship among non-redundant multilocus genotypes, revealing the genetic structure of our study system in space and time.

Given that we genotyped the same populations in two different points in time, we estimated the effective population size (*N*_e_) using the temporal method of Nei and Tajima (1981) and Waples (1989), as implemented in NeEstimator v.2 (Do *et al.* 2014). The *N*_e_ parameter is computed by relating the observed amount of temporal change in allele frequency to that expected under pure genetic drift. The comparison of *N*_e_ values among populations allows the inference of the extent of genetic drift in them, assuming that the effect of systematic forces, i.e. mutation, selection and migration, was low and that sampling effects were similar in all populations (Wang 2005). We used plan II, i.e. sampling before reproduction and not replaced, to estimate the standardized variance in the temporal change of allele frequency, which is reciprocally proportional to *N*_e_, by using all individuals from the two samplings per population and without restrictions on the lowest allele frequency. The 95 % CIs for *N*_e_ were estimated by the Jackknife method on loci.

Two flowering time genes (*FRI* and *FLC*) and one seed dormancy gene (*DOG1*) were sequenced in 21–23 haphazardly chosen individuals per population of the second sampling. We used existing sequence data on these genes from 9–10 individuals of the same populations from previous works corresponding to the first sampling (2003–04; Kronholm *et al.* 2012; Méndez-Vigo *et al.* 2013). These genes, known to account for variation in flowering time and seed dormancy in *A. thaliana*, were previously analysed in Iberian *A. thaliana* accessions and populations (Méndez-Vigo *et al.* 2011, 2013; Kronholm *et al.* 2012), allowing the identification of the gene regions concentrating the highest nucleotide diversity of interest. Thus, we sequenced the complete *FRI* gene (3.5 kb; Méndez-Vigo *et al.* 2011, 2013), a fragment corresponding to a 0.70-kb segment of *FLC* intron 1 (Méndez-Vigo *et al.* 2011, 2013) and a fragment corresponding to a 0.39-kb segment of *DOG1* exon 1 (Kronholm *et al.* 2012). In all cases, between one and seven overlapping fragments of 0.5–0.7 kb were PCR-amplified using described primers (Méndez-Vigo *et al.* 2011, 2013; Kronholm *et al.* 2012). PCR products were sequenced using an ABI PRISM 3700 DNA analyser (Applied Biosystems, Foster City, CA, USA). DNA sequences were aligned using DNASTAR v.8.0 (Lasergene, Madison, WI, USA). Alignments were inspected and edited by hand with GENEDOC v.2.7.0 (Nicholas *et al.* 1997). Nucleotide diversity was estimated with DnaSP v.5 (Librado and Rozas 2009). Polymorphisms were used to estimate the number and frequency of non-redundant multilocus haplotypes (*N*_H_) per gene, population and sampling. We did not use random subsamples of equal size to estimate gene parameters because variation in sample sizes was much lower in genes than in microsatellites. Besides, the ratio between the number of individuals from the first and second samplings used for gene sequencing was practically the same for all populations. GenBank accession numbers of DNA sequences generated in this study from the second sampling are MF142982–MF143073 for *FRI*, MF142894–MF142981 for *FLC* and MF142804–MF142893 for *DOG1*. Accession numbers of gene sequences from the first sampling are available elsewhere (Kronholm *et al.* 2012; Méndez-Vigo *et al.* 2013).

## Results

### Climatic trends

**Figure 2.**
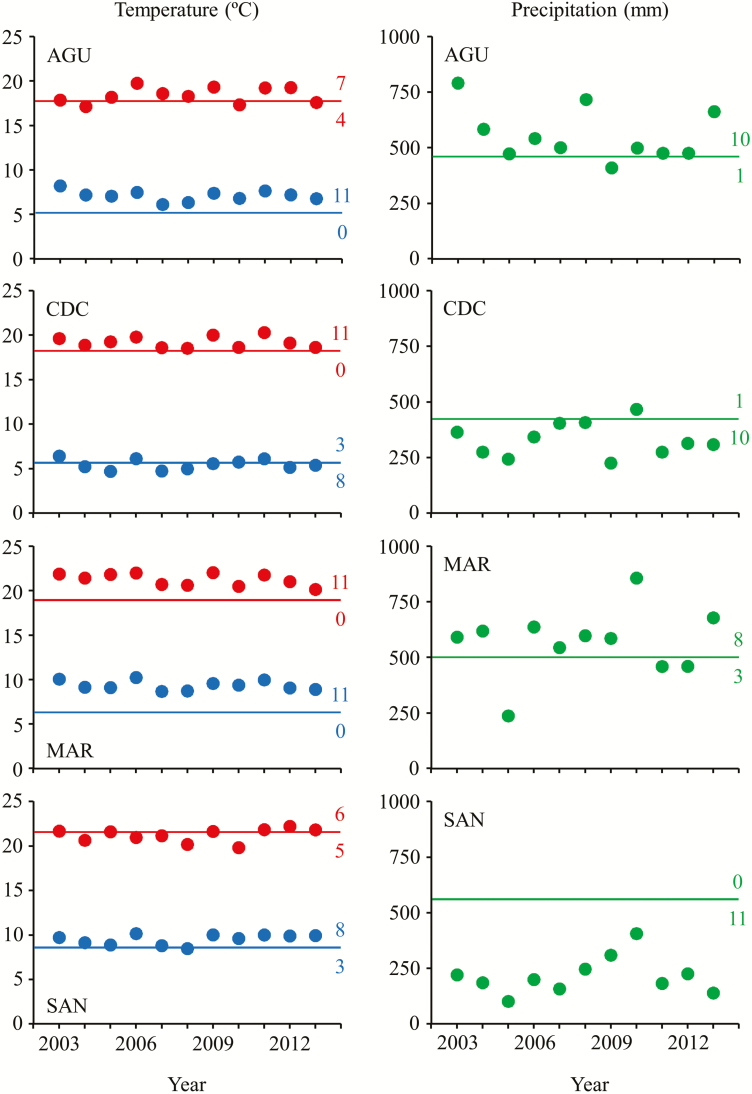
Average annual minimum temperatures (blue dots), average annual maximum temperatures (red dots) and annual total precipitation (green dots) for each *Arabidopsis thaliana* population for the period 2003–13. For each weather record, lines indicate the historical value corresponding to the period 1950–99, extracted from the Digital Climatic Atlas from the Iberian Peninsula. For the sake of clarity, the number of years above and below the historical records is also given.

For each population, we plotted average annual minimum temperature, average annual maximum temperature and total annual precipitation recorded between 2003 and 2013 against the historical values obtained from the Digital Climatic Atlas from the Iberian Peninsula. Average annual minimum temperatures were clearly above the historical record in AGU, MAR and SAN (average ± SE range of average annual minimum temperature above the historical record = 0.52 ± 0.18–3.15 ± 0.17 °C), but not in CDC (average annual minimum temperature below the historical record = 0.33 ± 0.18 °C; Fig. 2). In the case of average annual maximum temperatures, there was a trend for annual values to surpass the historical record in AGU, CDC and MAR (average ± SE range of average annual maximum temperature above the historical record = 0.52 ± 0.28–2.06 ± 0.22 °C), but not in SAN (average annual maximum temperature below the historical record = 0.35 ± 0.24 °C; Fig. 2). Finally, substantial year-to-year variation in total annual precipitation was detected in all populations over the study period. CDC and SAN exhibited precipitation values below the historical record in practically all years (average ± SE range of total annual precipitation below the historical record = 111.89 ± 23.91–329.53 ± 26.59 mm), whereas AGU and MAR showed the opposite trend (average ± SE range of total annual precipitation above the historical record = 65.69 ± 48.80–86.71 ± 37.56 mm; Fig. 2).

### Flowering time

**Figure 3.**
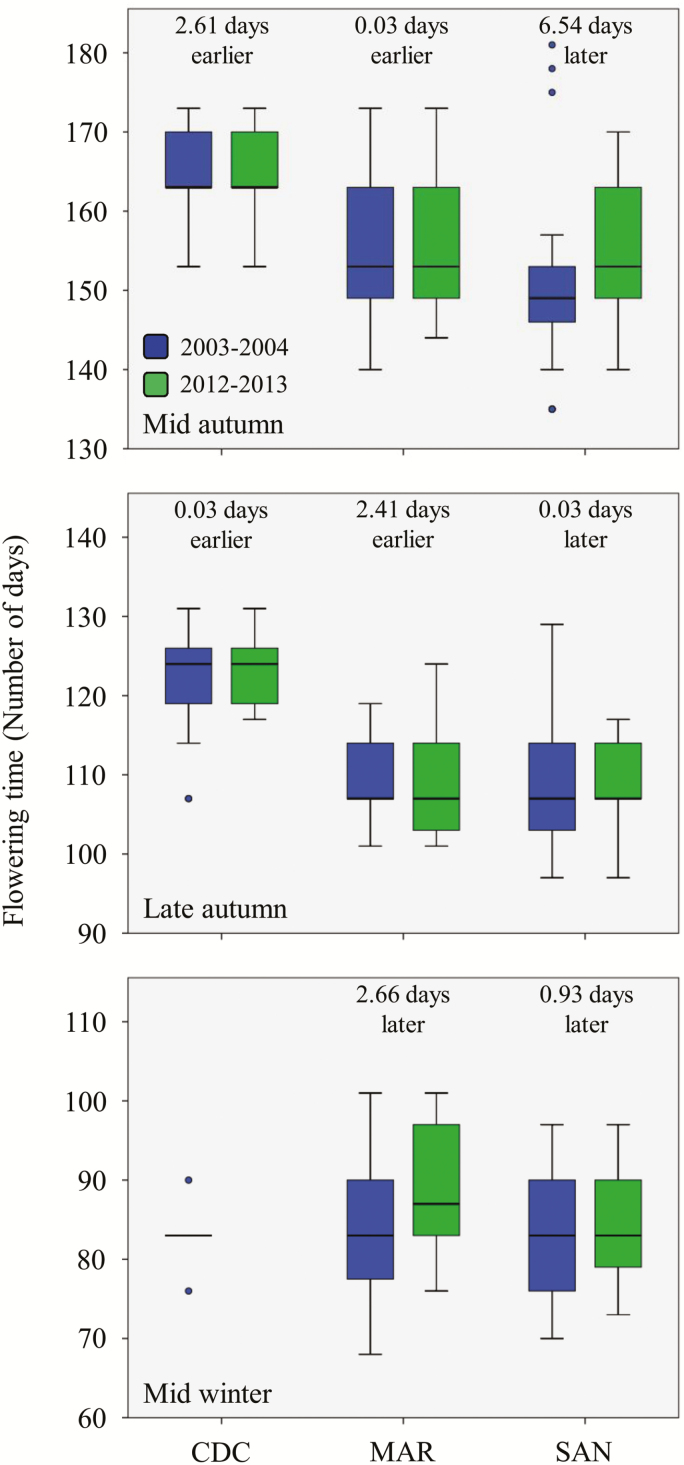
Summary statistics for flowering time. For each experiment and sampling, boxes show the lower and upper quartiles, whiskers are drawn down to the 10th percentile and up to the 90th, the line is the median of observations and hollow dots indicate outliers. For the sake of clarity, the mean difference in flowering time between samplings is also given. Only one CDC individual from the first sampling and none from the second sampling flowered in the mid winter experiment.

We conducted field experiments to quantify variation in flowering time of *A. thaliana* individuals between the two samplings. We carried out three sequential experiments, 2 months apart from each other, differing in sowing date, which forced all individuals to complete their life cycle in increasingly shorter periods of time. All individuals from all populations and samplings completed the life cycle in the mid autumn and late autumn sowing experiments. In the mid winter sowing experiment, all but one individual from CDC from the first sampling failed to complete the life cycle and died before reproduction (Fig. 3), only one individual from MAR from the first sampling did not reach maturity and all individuals from SAN from both samplings completed the life cycle.

Overall, our analyses indicated that sampling did not significantly affect flowering time in any experiment (Table 1). The mean (±SE) for flowering time was 157.2 ± 0.5, 113.5 ± 0.3 and 84.5 ± 0.4 days for the mid autumn, late autumn and mid winter experiments, respectively. Population and individuals nested within population were significantly different in nearly all experiments (Table 1). CDC was the population with the latest flowering times in all experiments (mean flowering time ± SE = 164.5 ± 0.6 and 123.1 ± 0.3 days for the mid autumn and late autumn experiments, respectively), followed by MAR (156.0 ± 0.9, 109.0 ± 0.5 and 86.0 ± 0.7 days for the mid autumn, late autumn and mid winter experiments, respectively) and SAN (152.8 ± 0.8, 109.0 ± 0.4 and 83.1 ± 0.5 days for the mid autumn, late autumn and mid winter experiments, respectively).

**Table 1.** General linear model testing the effect of sampling (2003–04 and 2012–13), population (CDC, MAR and SAN) and individuals nested within populations (14–15 individuals per population) on flowering time of *Arabidopsis thaliana* in three field experiments (sowing times in parenthesis). Degrees of freedom (*d.f.*), *F*-values and their significance are given for all factors and experiments. Significance: ***P < 0.0001; **P < 0.01; *P < 0.05; ns, non-significant.

Factor	Experiment #1 (mid autumn)		Experiment #2 (late autumn)		Experiment #3 (mid winter)	
	*d.f.*	*F*-value	*d.f.*	*F*-value	*d.f.*	*F*-value
Sampling (S)	1	1.28^ns^	1	0.70^ns^	1	2.94^ns^
Population (P)	2	5.27^ns^	2	46.08**	1	5.39*
Individual	42	3.64***	42	5.73***	28	4.95***
S × P	2	13.49***	2	7.47**	1	0.79^ns^
Error	299		475		256	

The interaction between sampling and population was also significant for the mid autumn and late autumn experiments (Table 1), indicating that the effect of sampling differed among populations. Differences between the two samplings for mean flowering time per population and experiment were small, ranging from a low of 0.03 to a high of 2.66 days, except for SAN in the mid autumn experiment with a difference of 6.54 days between samplings (Fig. 3). In CDC, individuals from the second sampling flowered earlier than individuals from the first sampling (Fig. 3). In the case of MAR, this population exhibited a similar behaviour, except for the mid winter experiment in which individuals from the second sampling flowered later than those from the first sampling (Fig. 3). Finally, SAN exhibited later flowering times for individuals from the second sampling than for individuals from the first sampling in all three experiments, particularly in the mid autumn experiment (Fig. 3).

Broad sense heritability (*h*^2^) values for flowering time for each population and experiment varied between 0.25 and 0.79 for individuals from the first sampling and between 0.00 and 0.65 for individuals from the second sampling (Table 2). Such a generalized decrease in *h*^2^ values for flowering time was mainly observed in SAN in all experiments, in MAR in the mid winter experiment and in CDC in the late autumn experiment (Table 2). Exceptions were CDC in the mid autumn experiment in which *h*^2^ values for flowering time slightly increased, and MAR in the two autumn experiments in which *h*^2^ values for flowering time remained rather similar (Table 2). SAN was the population with the most pronounced decrease in *h*^2^ values between samplings in all experiments (Table 2). In contrast, *h*^2^ values for flowering time in MAR showed the least difference between samplings, particularly in the mid and the late autumn experiments (Table 2).

**Table 2.** Broad sense heritability (*h*^2^) values for flowering time in three *Arabidopsis thaliana* populations estimated in three field experiments with individuals collected from two samplings (2003–04 and 2012–13). The 95 % CIs for *h*^2^ values are given in parentheses. No individuals from CDC, except one from the first sampling, were able to complete the life cycle in the mid winter experiment.

Population	Experiment	2003–04	2012–13
CDC	Mid autumn	0.25 (0.14–0.33)	0.39 (0.30–0.46)
CDC	Late autumn	0.79 (0.73–0.82)	0.08 (0.02–0.13)
CDC	Mid winter	–	–
MAR	Mid autumn	0.67 (0.60–0.72)	0.65 (0.57–0.69)
MAR	Late autumn	0.61 (0.53–0.66)	0.60 (0.53–0.65)
MAR	Mid winter	0.75 (0.69–0.79)	0.58 (0.50–0.63)
SAN	Mid autumn	0.76 (0.71–0.80)	0.33 (0.24–0.39)
SAN	Late autumn	0.67 (0.61–0.72)	0.00 (0.00–0.00)
SAN	Mid winter	0.32 (0.24–0.39)	0.19 (0.11–0.25)

### Genetic diversity

We used genetic data from a total of 329 *A. thaliana* individuals based on 12 nuclear microsatellite markers, including 103 individuals from the first sampling and 226 individuals from the second sampling, to assess temporal changes in genetic diversity, genetic differentiation and genetic structure in *A. thaliana* populations. After correcting for sample size differences between samplings and populations by random subsampling of equal size (*N* = 20), our results showed contrasting and pronounced patterns of temporal changes in genetic diversity parameters in *A. thaliana* populations. For example, the genetic diversity (*H*_S_) in AGU and MAR increased by 103.2 and 13.1 % between the two samplings, respectively (Table 3). In contrast, genetic diversity in CDC decreased by 19.8 %, whereas that in SAN remained fairly stable over time with a slight decrease of 7.9 % between the two samplings (Table 3). Observed heterozygosity (*H*_O_) and outcrossing rates (*O*_R_) substantially increased in all populations over the study period, except those in AGU that showed the opposite pattern (Table 3). Nonetheless, observed values for *H*_O_ and *O*_R_ in both samplings fell within the expected values for the highly self-fertilizing *A. thaliana*: *H*_O_ varied between 0.004 and 0.089, and *O*_R_ between 0.004 and 0.094 (Table 3).

**Table 3.** Genetic diversity of *Arabidopsis thaliana* populations obtained from each sampling estimated from 12 nuclear microsatellite loci. The number of multilocus genotypes (*N*_G_) with the number of sampled individuals in parenthesis (*N*), observed heterozygosity (*H*_O_), outcrossing rates (*O*_R_) and mean gene diversity (*H*_S_) are given. *H*_O_, *O*_R_ and *H*_S_ are means (±SD) from 100 subsamples of 20 individuals each per population and sampling.

Population	*N*_G_ (*N*)	*H*_O_	*O*_R_	*H*_S_
AGU (2004)	12 (20)	0.046 ± 0.000	0.094 ± 0.000	0.282 ± 0.000
AGU (2012)	31 (53)	0.021 ± 0.009	0.018 ± 0.008	0.573 ± 0.022
CDC (2003)	26 (32)	0.021 ± 0.008	0.020 ± 0.008	0.602 ± 0.015
CDC (2012)	38 (58)	0.066 ± 0.023	0.082 ± 0.038	0.483 ± 0.050
MAR (2003)	20 (30)	0.026 ± 0.008	0.033 ± 0.014	0.540 ± 0.039
MAR (2012)	63 (70)	0.089 ± 0.022	0.068 ± 0.018	0.611 ± 0.018
SAN (2003)	18 (21)	0.004 ± 0.001	0.004 ± 0.001	0.671 ± 0.008
SAN (2013)	22 (45)	0.019 ± 0.009	0.017 ± 0.009	0.618 ± 0.032

**Figure 4.**
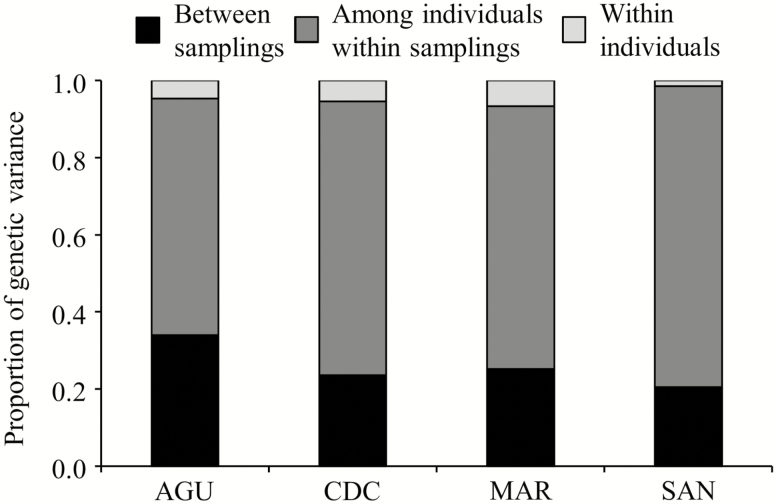
Proportion of genetic variance obtained from AMOVA for each *Arabidopsis thaliana* population. Genetic variance is partitioned between samplings, among individuals within samplings and within individuals. Mean values from 100 analyses using subsamples of equal size are given. Standard deviations are not shown because they were very low.

The AMOVA indicated that the extent of genetic differentiation between the two samplings was substantial in all populations, with values as high as 34.0 % for AGU, 23.6 % for CDC, 25.1 % for MAR and 19.9 % for SAN (Fig. 4). These results indicated that the highest temporal genetic differentiation values were observed in the northern and central populations (AGU, CDC and MAR), whereas the lowest value was recorded in the southernmost population (SAN). Genetic differentiation among individuals within samplings reached the highest values, ranging between 61.4 % (AGU) and 76.0 % (SAN) (Fig. 4). As expected for a highly self-fertilizing plant, genetic differentiation within individuals showed the lowest values (range = 1.4–6.7 %; Fig. 4). When taken all data together in a single AMOVA including sampling as the highest hierarchical level, we found that genetic differentiation between samplings was of 3.1 %, genetic differentiation among populations within samplings of 29.9 %, genetic differentiation among individuals within populations of 63.0 % and genetic differentiation within individuals of 4.0 %. All *F*-statistics were significant (*P* < 0.0001).

**Figure 5.**
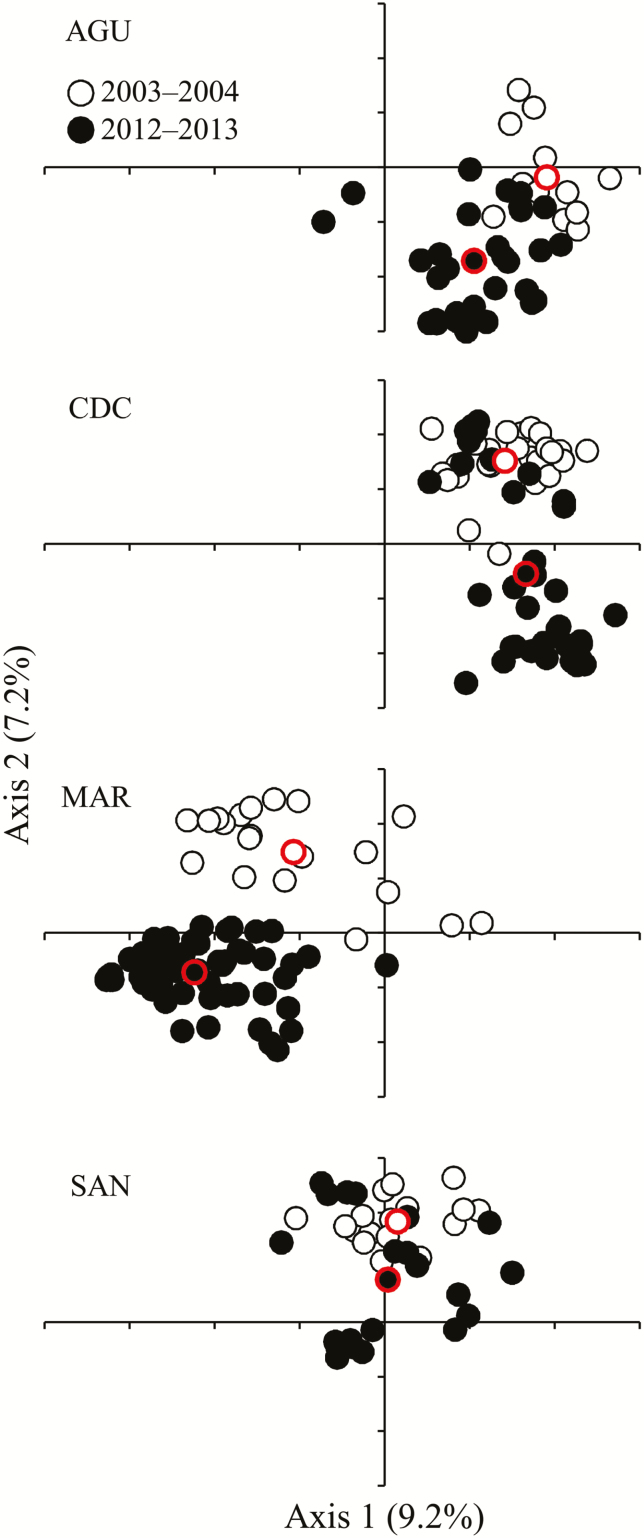
Scatter plots displaying the first two eigenvectors estimated by PCoA of *Arabidopsis thaliana* non-redundant multilocus genotypes using 12 nuclear microsatellites. For the sake of clarity, populations are shown in different panels, although eigenvectors come from a unique analysis combining all data from all populations and samplings. Multilocus genotypes from the first sampling (2003–04; hollow dots) and multilocus genotypes from the second sampling (2012–13; filled dots) are indicated for each population. The centroids (dots with red contour) for multilocus genotypes from the two samplings and populations are also indicated.

We found that 69.9 % of the genotyped *A. thaliana* individuals (230 of 329) were non-redundant multilocus genotypes (Table 3). Non-redundant multilocus genotypes represented by more than one individual (48 of 230; 20.9 %) included a minimum of two individuals and a maximum of 14 (mean ± SD = 3.1 ± 2.2 individuals per multilocus genotype). It must be noted that no identical multilocus genotype was found among populations or between samplings. All individuals with the same multilocus genotype came from the same population and sampling. Non-redundant multilocus genotypes from all populations and samplings were also used to explore how the genetic structure of *A. thaliana* populations varied between samplings by means of PCoA. The first three eigenvalues explained up to 22.4 % of the variation. The graphical representation of the first two axes, accounting for 16.4 % of the variation, indicated a clear trend for temporal structuring between multilocus genotypes from the two samplings in each *A. thaliana* population (Fig. 5). MAR exhibited the clearest pattern of temporal genetic structuring between samplings (distance between centroids for multilocus genotypes from the first and second samplings = 0.50), AGU and CDC exhibited intermediate overlap patterns between multilocus genotypes from the two samplings (0.36 and 0.41 for AGU and CDC, respectively) and SAN showed the highest overlap between multilocus genotypes from the two samplings (0.20; Fig. 5). Thus, SAN had the largest genetic similarities between multilocus genotypes detected in the two samplings (Fig. 5).

We evaluated the extent of genetic drift in all populations by estimating effective population size (*N*_e_) with the temporal method. AGU exhibited a *N*_e_ value of 10.8 (95 % CI = 7.3–15.3). The rest of populations had higher *N*_e_ estimates with values of 18.4 (12.8–25.4) for CDC, 20.5 (12.5–31.1) for MAR and 21.5 (16.0–28.3) for SAN. Hence, the effects of genetic drift in CDC, MAR and SAN were rather similar, whereas those in AGU were more intense. This result can be related to the fact that the lowest genetic diversity (*H*_S_) was in AGU in the 2004 sampling (Table 3).

We also sequenced the regions concentrating the highest nucleotide diversity in two flowering time genes (*FRI* and *FLC*) and one seed dormancy gene (*DOG1*) in individuals from the two samplings from each population. *FRI* was the gene with more polymorphisms in all populations (range = 2–20 single nucleotide polymorphisms [SNPs]) generating more multilocus haplotypes (Table 4). *FLC* and *DOG1* exhibited less polymorphisms or even none at all (range = 0–4 SNPs and 0–8 SNPs for *FLC* and *DOG1*, respectively; Table 4). Temporal patterns of variation in silent nucleotide diversity were quite erratic in these genes: *FRI* decreased its nucleotide diversity in all populations except in AGU, *FLC* decreased it in AGU and CDC and increased it in MAR and *DOG1* sharply decreased it in AGU but strongly increased it in MAR and SAN (Table 4).

**Table 4.** Genetic diversity of *Arabidopsis thaliana* populations from each sampling estimated for two flowering genes (*FRI* and *FLC*) and one seed dormancy gene (*DOG1*). The number of multilocus haplotypes (*N*_H_) with the number of sampled individuals in parenthesis (*N*), the number of polymorphisms (NP) that were not in linkage disequilibrium, and silent (π_silent_) and non-synonymous (π_non-syn_) nucleotide diversity are given.

Population	*FRI*				*FLC*				*DOG1*			
	*N*_H_ (*N*)	NP	π_silent_	π_non-syn_	*N*_H_ (*N*)	NP	π_silent_	π_non-syn_	*N*_H_ (*N*)	NP	π_silent_	π_non-syn_
AGU (2004)	2 (9)	5	0.00076	0.00055	4 (9)	4	0.00454	–	3 (10)	3	0.00595	0.00374
AGU (2012)	2 (23)	5	0.00147	0.00042	2 (23)	3	0.00080	–	2 (23)	2	0	0.00292
CDC (2003)	5 (9)	12	0.00204	0.00094	4 (9)	3	0.00232	–	1 (10)	0	0	0
CDC (2012)	3 (23)	2	0.00012	0	3 (23)	3	0.00217	–	1 (23)	0	0	0
MAR (2003)	6 (9)	12	0.00373	0.00153	3 (9)	2	0.00107	–	3 (10)	5	0.00395	0.00148
MAR (2012)	15 (23)	16	0.00255	0.00158	5 (21)	4	0.00256	–	4 (21)	5	0.03650	0.00370
SAN (2003)	5 (9)	13	0.00163	0.00248	1 (9)	0	0	–	4 (10)	7	0.00224	0.00560
SAN (2013)	9 (23)	20	0.00149	0.00199	1 (21)	0	0	–	7 (23)	8	0.01108	0.00550

## Discussion

The analysis of phenotypic and genetic variation between individuals of the same populations sampled at different times enables us to quantify how populations have actually changed across generations. Annual plants are particularly well suited for conducting the resurrection approach, as shown by recent illuminating examples on *A. thaliana* (Frachon *et al.* 2017), *Brassica rapa* (Franks *et al*. 2007, 2016; Franks and Weis 2008; Welt *et al.* 2015), *Centaurea cyanus* (Thomann *et al.* 2015), *Datura stramonium* (Bustos-Segura *et al.* 2014), *Hordeum spontaneum* (Nevo *et al.* 2012), *Ipomoea purpurea* (Kuester *et al.* 2016), *Polygonum cespitosum* (Sultan *et al.* 2013; Horgan-Kobelski *et al.* 2016) and *Triticum dicoccoides* (Nevo *et al.* 2012). All these resurrection studies certified how fast annual plant populations can change over relatively short periods of time as a result of various environmental pressures.

We applied a resurrection approach on *A. thaliana* by sampling four Spanish natural populations twice over a decade. Aerial orthophotographs indicated that populations did not experience major disturbances over the study period **[see****Supporting Information—Fig. S1****]**. Based on that, we assume that temporal variation in phenotypic and genetic attributes chiefly responded to the variation in the climatic conditions, although we ignore how other biotic factors, such as herbivores or pathogens, might have affected these populations. Based on the comparison between annual weather records over the study period and historical records, we detected a trend for warming in all populations over the study period when compared to their historical temperature records (Fig. 2). Only CDC exhibited more years with average annual minimum temperature slightly below the historical record for that population. Overall, this trend is in agreement with the warming patterns predicted to occur in the region throughout the 21st century (Gómez-Navarro *et al.* 2010). In contrast, total annual precipitation exhibited a different pattern, with two populations above and other two below their respective historical records over the study period. This result also supports the accepted view that regional-scale climate change projections for precipitation still remain largely uncertain (Christensen *et al.* 2008; Schaller *et al.* 2011; Mahlstein *et al.* 2012; Tsanis *et al.* 2013), which is a problem to figure out the actual climatic scenarios for the near future. Hence, the four *A. thaliana* populations represented two distinct climatic scenarios: intense warming with increased precipitation, i.e. AGU and MAR, and moderate warming with decreased precipitation, i.e. CDC and SAN.

Our field experiments, designed to quantify differentiation in flowering time in *A. thaliana* over time, indicated that mean flowering time of *A. thaliana* populations did not differ substantially between samplings. However, the interaction between sampling and population was significant for the first two experiments in mid and late autumn including all populations. In CDC and MAR, *A. thaliana* advanced flowering with time, whereas in SAN the species exhibited the opposite behaviour, particularly in the mid autumn experiment where individuals from the second sampling flowered on average 6.5 days later than those from the first sampling (Table 1). A recent resurrection approach on *A. thaliana* encompassing 8 years also found a mean delay of 6.1 days in bolting time in a French population that experienced a significant warming of more than 1 °C over the last 30 years (Frachon *et al.* 2017). Although it is difficult to provide an explanation for these patterns, such a great difference in flowering time in SAN between the two samplings was only evident in the mid autumn experiment, when individuals experienced the longest vegetative phase prior to reproduction. This result stresses the important effect that the environment experienced by individuals during their development may have on key life-history traits (Donohue 2005, 2009, 2014), as well as the enormous complexity of the genotype × environment interaction in the expression of flowering time in *A. thaliana* (Stratton 1998; Méndez-Vigo *et al.* 2013, 2016; El-Soda *et al.* 2014; Sasaki *et al.* 2015; Ågren *et al.* 2017).

Beyond the trends observed for mean flowering time, broad sense heritability (*h*^2^) values for flowering time did show a more meaningful pattern of variation between individuals from the two samplings. In particular, there was a trend for reduction in *h*^2^ values for flowering time in populations from the second sampling (Table 2). This result indicated that the amount of among-individual variance in flowering time shrank over the study period. We hypothesize that environmental variation over the study period might have imposed stabilizing selection on flowering time, reducing therefore among-individual variation in this trait, although the exact mechanisms underlying stabilizing selection on flowering time cannot be determined from these data. When looking at the two extreme populations in terms of mild and sharp reduction in *h*^2^ values for flowering time, MAR and SAN, respectively, we speculate that the scenario of moderate warming and decreased precipitation in SAN would have stronger effect on *h*^2^ values for flowering time than intense warming and increased precipitation in MAR. Hence, it is plausible to consider drought stress as a major force in determining plant response and population performance in *A. thaliana*, as shown by various studies quantifying the effects of drought on a suite of *A. thaliana* traits dealing with life history, physiology, resource allocation and resistance to herbivores (McKay *et al.* 2003; Rizhsky *et al.* 2004; Riboni *et al.* 2013; Wolfe and Tonsor 2014; El-Soda *et al.* 2014; Zhang *et al.* 2015; Davila Olivas 2017*a*, *b*; Exposito-Alonso *et al.* 2018*b*). However, further work will be needed in order to test this hypothesis by increasing the number of populations resampled in two points in time and differing in the degree of warming and precipitation intensity.

From a genetic viewpoint, *A. thaliana* populations also showed distinctive genetic changes over the study period. As far as neutral microsatellites are concerned, AGU and MAR increased their genetic diversity between samplings, CDC decreased it and SAN barely maintained the same value (Table 3). It is worth noting that the two populations with intense warming and increased precipitation, i.e. AGU and MAR, where also those that showed increased genetic diversity over time. This result highlights the important role that precipitation patterns may have for the genetic make-up of *A. thaliana* populations affected by different warming intensities. Although this conclusion seems plausible, we must assume that these changes probably reflect natural demographic fluctuations affecting allele frequencies over time. This assumption is supported by the effective population size (*N*_e_) estimates indicating that the effects of genetic drift on these populations were rather similar, except for AGU that showed the lowest *N*_e_ value.

This demographic scenario would also be applicable to the temporal patterns of variation in silent nucleotide diversity of flowering time and seed dormancy genes, which were rather erratic and gene dependent, probably as a result of the lower number of polymorphisms found in these genes (Table 4). Despite the fact that we also detected non-synonymous substitutions, all of them, except a FRI truncation only detected in AGU in the first sampling, were not associated to phenotypic changes in flowering time (Méndez-Vigo *et al.* 2011, 2013) or seed dormancy (Kronholm *et al.* 2012). This supports the assumption that changes detected in these flowering time and seed dormancy genes are probably determined by natural demographic fluctuations. Although these genes underlie major quantitative trait loci for flowering time and seed dormancy, it is clear that understanding the spatio-temporal variation in the genetic basis of these polygenic key life-history traits in natural populations is still an outstanding question in plant biology.

In contrast, genetic differentiation and genetic structure analyses did provide meaningful results when comparing the same populations between the two samplings. For example, genetic differentiation between samplings remarkably differed from a low of 20 % to a high of 34 % in just up to a decade. This is in agreement with other resurrection studies in annuals reporting substantial genetic changes in plant populations over decades (Vigouroux *et al.* 2011; Nevo *et al.* 2012; Van Dijk and Hautekèete 2014; Kuester *et al.* 2016). Based on this result, it can be concluded that natural *A. thaliana* populations exhibited a quite dynamic demographic behaviour, which is known to be characterized by dramatic year-to-year variation in plant abundance (Picó 2012) and a relatively short seed permanency in the soil seed bank (2–4 years; Lundemo *et al.* 2009; Montesinos *et al.* 2009; Falahati-Anbaran *et al.* 2014; Postma *et al.* 2016), features that easily lead to rapid temporal genetic differentiation. Besides, *A. thaliana* is known to exhibit low inter-population migration rates and limited dispersal distances (Lundemo *et al.* 2009; Bomblies *et al.* 2010; Falahati-Anbaran *et al.* 2014), which minimizes the effect of gene flow on genetic differentiation over *A. thaliana* generations. Thus, at this particular local scale and for non-urban populations, it is well accepted that genetic variation in *A. thaliana* is mostly accounted for by novel mutations, outcrossing and recombination, enhanced by the species’ low linkage disequilibrium, that altogether have the potential to generate genetic novelty in *A. thaliana* in a relatively short period of time (Bomblies *et al.* 2010; Gomaa *et al.* 2011).

In this resurrection study, we have been able to quantify the actual impact of all the processes mentioned above on natural *A. thaliana* populations over time. The best picture of the genetic temporal change in *A. thaliana* was given by the genetic structure between samplings based on non-redundant multilocus genotypes. SAN and MAR were the populations with the lowest and the highest genetic divergence over the study period, respectively. Interestingly, the two most different populations, in terms of warming, precipitation patterns and broad sense heritability values for flowering time, were also the ones with the most different genetic structuring over time. In conclusion, we hypothesize that dramatically drier conditions, such as those experienced in SAN over the study period, would have the potential to homogenize *A. thaliana* populations by reducing among-individual variation in key quantitative traits as well as by constraining genetic differentiation over time. It must be noted that this trend does not come into conflict with maintaining demographically viable populations with high levels of microsatellite and gene genetic diversity, as occurred in SAN and MAR. Hence, these results might illustrate the ability of Iberian *A. thaliana* populations to cope with harsher environmental conditions imposed by climate change (see experimental evidence in Exposito-Alonso *et al.* 2018*a*).

It remains to be seen whether the significant and dynamic phenotypic and genetic changes detected in *A. thaliana* populations in just a decade affect the long-term population persistence under a scenario of increasing warming with increased or decreased precipitation. However, we want to stress the importance of quantifying the pace and intensity of temporal change in plant populations by resampling populations periodically and conducting resurrection experiments. In the case of *A. thaliana*, we need to scale up the study presented here to a larger number of *A. thaliana* populations across different environments in the Iberian Peninsula from which we possess seed collected and preserved since early 2000s. Recently, we have shown that warm and cool Iberian environments might be exerting very different selective pressures on both flowering time and seed dormancy, but also on the correlation between the two traits in *A. thaliana* (Vidigal *et al.* 2016; Marcer *et al.* 2018). Thus, resurrection experiments dealing with warm and cool Iberian populations would provide a proof of concept of the microevolutionary implications of climate change in annual and short-lived plants.

## Data

Data available from the Dryad Digital Repository: https://doi.org/10.5061/dryad.84vc24v.

## Sources of Funding

F.X.P., A.M. and R.G. were funded by grants CGL2012-33220/BOS and CGL2016-77720-P (AEI/FEDER, UE). A.M. acknowledges the Agency for Management of University and Research Grants of the Generalitat de Catalunya (2014-SGR-913).

## Contributions by the Authors

F.X.P. and C.A.-B. planned and designed the research. R.G., B.M.-V., A.M., C.A.-B. and F.X.P. conducted field work, performed experiments and/or analysed data. F.X.P. wrote the first draft of the manuscript and all authors contributed to the final version of it.

## Conflict of Interest

None declared.

## Supplementary Material

Supplementary Figure S1

## Supporting Information

The following additional information is available in the online version of this article—

**Figure S1.** Aerial orthophotographs of *Arabidopsis thaliana* populations. Orthophotographs were chosen from those available to the closer years for the first and second samplings. The circular area (500 m radius) around the GPS coordinate is indicated, which was previously used to describe the ecological characteristics of populations.
