# Supplementary material for: Quantifying temporal change in plant population attributes: insights from a resurrection approach
Source: AoB Plants. 2018 Oct 10;10(5):ply063. doi: 10.1093/aobpla/ply063 (PMC6198925; doi:10.1093/aobpla/ply063)
Supplement: Supplementary Figure S1 [file ply063_suppl_supplementary_figure.pdf]

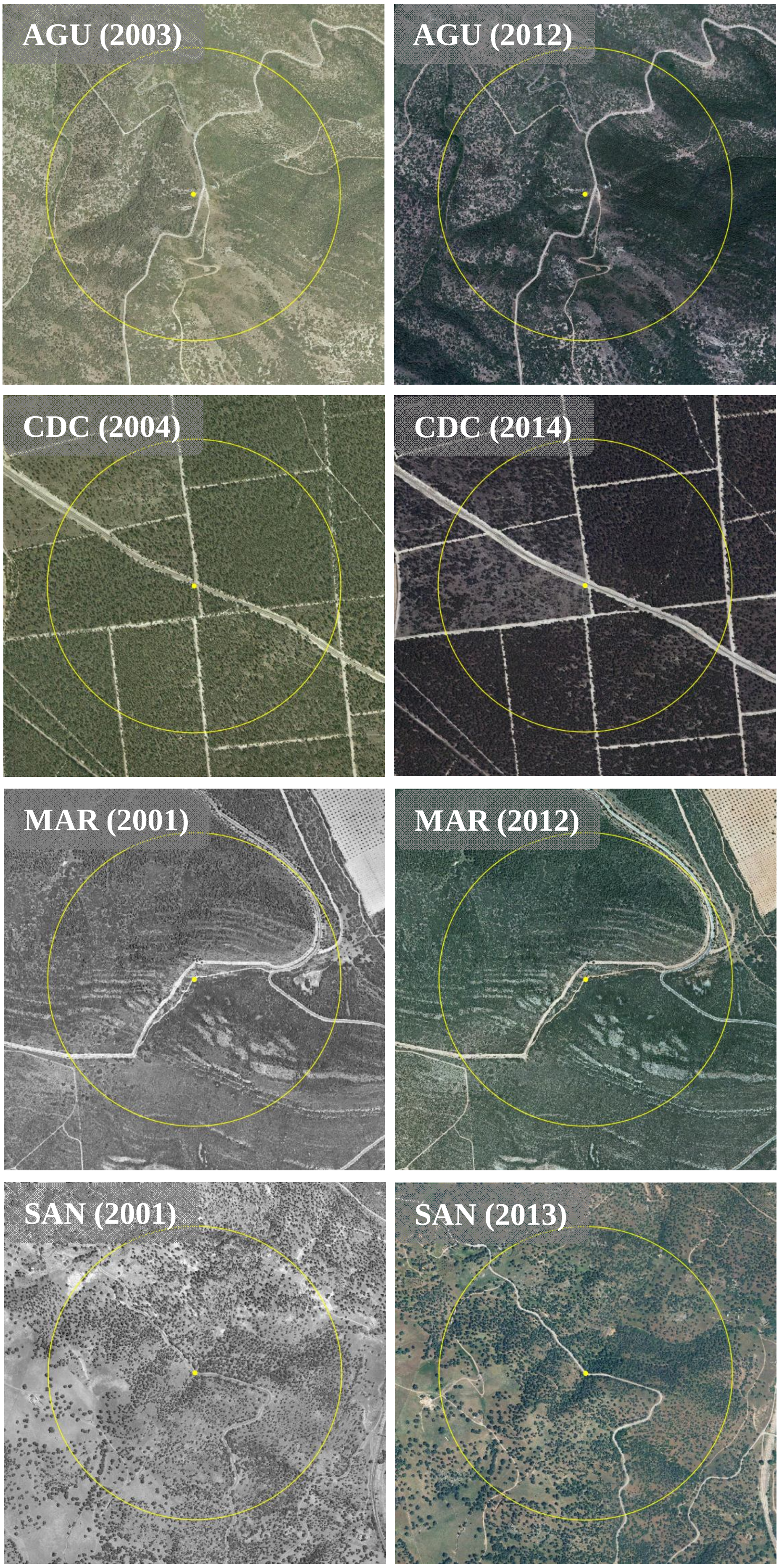

Figure S1. Aerial orthophotographs of *Arabidopsis thaliana* populations. Orthophotographs were chosen from those available to the closer years for the first and second samplings. The circular area (500 m radius) around the GPS coordinate is indicated, which was previously used to describe the ecological characteristics of populations.
